# The effect of different imaging techniques for the visualisation of evidence in court on jury comprehension

**DOI:** 10.1007/s00414-019-02221-y

**Published:** 2019-12-06

**Authors:** D. Errickson, H. Fawcett, T. J. U. Thompson, A. Campbell

**Affiliations:** 1 Cranfield Forensic Institute, Defence Academy of the United Kingdom, Cranfield University, Shrivenham, SN6 8LA UK; 2 Department of Psychology, Manchester Metropolitan University, Manchester, M15 6BH UK; 3 School of Health and Life Sciences, Teesside University, Borough Road, Middlesbrough, TS1 3BA UK

**Keywords:** Courtroom, 3D imaging, 3D printing, Bias, Perception, Decision-making, Photography

## Abstract

Evidence presented within a courtroom should be clear so that the members of the jury can understand it. The presentation of distressing images, such as human remains, can have a negative effect on the jury since photographic images may evoke emotional responses. Therefore, it is important to understand how other visual mediums may improve comprehension, bias, or distress individuals. For this study, 91 individuals were randomly assigned one of three visual evidence formats in a mock courtroom exercise. These included photographs, 3D visualisations, or a 3D-printed model. The results show that the use of 3D imaging improves the juror’s understanding of technical language used within a courtroom, which in turn better informs the juror’s in their decision-making.

## Introduction

The unbiased presentation and interpretation of data in the courtroom has been emphasised as one of the main issues in forensic science [1]. Understanding the evidence shown in a courtroom is important [2], and therefore, it should be presented in a way that a judge and jury are able to comprehend it [3]. The use of evidence in a courtroom will always generate debate on how the information should be visualised and presented in a meaningful way [4]. This debate is often fuelled by juror’s unrealistic expectations of legislative formats and available technology, and if not considered, may negatively impact their decision-making [5, 6].

This problem is exacerbated with regard to skeletal trauma evidence as human remains should not be taken into the courtroom [7]. Moreover, this physical evidence may be reburied before the case comes to trial. Photographs are a standard recording tool in forensic science that can be examined long after the physical evidence and is therefore used as an aid of explaining the information [8]. This is advantageous because photographic information can help the jurors comprehend the point of discussion.

Three-dimensional imaging, virtual environments, and simulations are now becoming commonplace in the reconstruction and documentation of crime scenes, artefacts, and skeletal evidence [9–16]. Ampanozi et al. [17] demonstrated that the use of 3D reconstructions and colour-coded CT images were a preferred visual format for district attorneys for understanding radiological findings. Further, Blau et al. [18] have demonstrated that the comprehension of verbal presentations improved with the use of visual aids. Therefore, this study was designed to investigate whether the use of visualisation techniques influenced the juror’s verdict and whether the visual format of the evidence improved the understanding of technical language within the court case.

## Materials and methods

A script was created to mimic that of a courtroom trial. The scenario detailed two adult males leaving a public house. On their way home, one of the individuals survived a fall that had resulted in a severe head injury associated with a cranial fracture. The mock trial was created so that jurors could determine whether the victim fell or was pushed by the defendant. The script was read by voice actors who were used to play the prosecution, defence, judge, usher, and witnesses including the forensic anthropological expert witness. This recording was played to the jury, who also had access to the script in front of them.

**Fig. 1:**
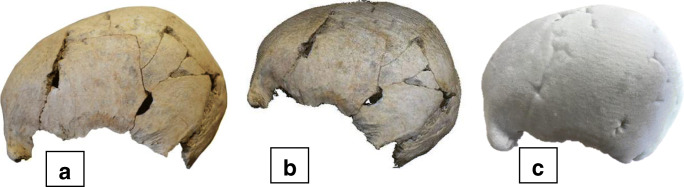
A photograph (**a**), 3D reconstruction (**b**), and 3D-printed model (**c**) of the cranium from Stanground South, Peterborough [19]

The forensic expert presented evidence about the head trauma sustained by the victim. For the cranium of the victim, an example from the archaeological excavation at Stanground South, Peterborough, was utilised [19]. Although a limitation to this study, an archaeological cranium was used because of the traumatic visual characteristics and availability. This was photographed using a Nikon D3100, and a 3D model was created using the PicoScan (3DDynamics, Belgium) structured light scanning method. The resulting 3D digitisation was then 3D printed using a ZCorporation 450 (USA) using CA-Bond PC08 Cyanoacrylate (Fig. 1). These three visual techniques made up the three different demonstrative evidence variables and were created using the standards developed by Errickson and colleagues [20].

A full audio script of the mock trial was played to the mock jury. Jurors were a mix of jury-eligible students from Teesside University and volunteers from the local area. Traditionally, jury decision-making research has used college students [23]. Further, as demonstrated by Bornstein et al. [22], the use of student mock jurors has very limited variance against the decision-making of actual jurors. Therefore, the use of students in this study is not a limitation. The full audio script remained the same throughout the experiment and participants were not told about the context of the study until after the experiment to ensure the results were not biased. Providing the participants with no contextual information prior to the study did not create any health risks to the participants and is standard in these types of studies. Several sessions took place with no more than 20 jurors at one given time. In each session, the audio script was played to the mock jurors, and the participants in that particular session were randomly assigned one of the three imaging techniques for visualisation when the expert witness discussed their findings. For the photographic images and the 3D model, these images were displayed real time to the jurors in the form of a video demonstration, and they matched up to the particular area that the forensic pathologist was discussing. With regard to the 3D-printed model, this was physically demonstrated to the jury as an object to be passed between them.

After the mock trial, jurors were independently given a questionnaire which requested the participants decision about whether the victim had been pushed or accidently fallen. The questions included their opinion on verdict, their confidence and reasoning for their verdict, the clarity of the evidence displayed, and their understanding of the technical language used throughout the trial. For the confidence- and clarity-based questions, the participants marked their opinion on a 100-mm scale (e.g. a mark at 100 mm gave 100% confidence). This was later converted into a percentage for quantitative analysis using the Statistical Package for Social Sciences (SPSS).

For this study, full ethical approval was obtained through Teesside University’s ethics committee (Ethical Approval Number 992). Upon completion of the experiment, full disclosure of the entire study was declared.

## Results

Ninety-one (82%) of the 111 participants who took part in the study fully completed their questionnaire and the results were incorporated in to the final analysis. The 20 questionnaires that were excluded from this research were due to the failure of the individual to sign the consent form. Although there were no comparisons made between males and females, the overall male to female ratio of participants was 28/63. Due to the accessibility of participants, 70% of mock jurors were between 18 and 21 years old. Of the varying visual presentations, 24 participants (26.3%) were randomly assigned the photographic images, 33 participants (36.3%) assigned the 3D printed models, and 34 participants (37.4%) assigned the 3D digitisations. Unfortunately, due to some of the questionnaires being incomplete, the total number of participants for each visual modality was not the same.

### The overall verdict

Regarding the overall verdict of the findings, there was no significant correlation between the results (Table 1). Further, the jurors were asked to state their confidence with regard to their verdicts (Table 2). Mann-Whitney tests were performed between the visualisation types to test whether the jurors had a high state of confidence depending on the visualisation technique shown. Again, there was no significance, although the *p* value between 3D digitisations and 3D-printed models was 0.061.

**Table 1:** The total count for the juror’s verdict (guilty or not guilty) for each of the visualisation techniques and the overall mean rank

Visualisation type	Not guilty	Guilty	Total	Kruskal-Wallis mean rank
Photographic imaging	18	6	24	49.13
3D digitisations	26	8	34	49.79
3D-printed models	18	15	33	39.82
Total	62	29	91	*p* value = 0.113

**Table 2:** The significance of confidence for each visualisation type using a Mann-Whitney *U* test

Mann-Whitney test		*p* value	Mann-Whitney test		*p* value	Mann-Whitney test		*p* value
Photograph	32.38	0.117	Photograph	29.25	0.898	3D digitisations	37.62	0.061
3D-printed models	26.55		3D digitisations	29.68		3D-printed models	30.27	

In addition to the verdict, the participants could detail the reason behind their decision by free text. Interestingly, where the “not guilty” verdict was given, 37 (60% of not guilty judgements) gave their reasoning as, “insufficient evidence” within the trial. A large proportion of the remaining jurors (21/62) stated that their overall reason for a “not guilty” verdict was based on the “victim” having an “impaired judgement” (34%). On the other hand, the proportion of guilty verdicts was reasoned to be due to the “force required” to break the cranium (62% of all guilty verdicts). Other reasons stated included the relationship between the two individuals, the doubt displayed in the expert witnesses’ voice, and that the defence may be lying.

### Evidence comprehension

The clarity of images was assessed by a juror’s mark on a 100-mm line. Overall, most of the jurors found the images comprehensible, and on average, photographs (74%) and 3D digitisations (73%) had a similar overall clarity with 3D-printed models (78%) being slightly more comprehensible. This showed no significance in correlation (*p =* 0.784). Likewise, a Kruskal-Wallis test also demonstrated no significance between the comprehension of the evidence and the verdict given (Table 3).

**Table 3:** Displaying the Kruskal-Wallis significance with regard to image clarity against the juror’s verdict

Verdict	Kruskal-Wallis mean rank	*p* value
Guilty	45.26	0.855
Not guilty	46.35	

### Technical language

Although the statistical test demonstrates no significance, the juror’s understanding of the technical language increased with the use of 3D visualisations (Table 4). For example, for the photographic images, 79% of jurors stated they understood the technical language. The understanding of the technical language further increased with the use of 3D digitisations (88% of jurors stated they understood) and 3D-printed models (94% of jurors stated they understood the language).

**Table 4:** The table demonstrates the total count for whether the jurors understood the technical language or not for each of the visualisation groups

Visualisation Type	Understood language	Did not understand language	Kruskal-Wallis mean rank
Photographic imaging	19	5	49.98
3D digitisations	30	4	45.85
3D-printed models	31	2	43.26
Total	80	11	*p* value = 0.243

### Overall significance

Table 5 demonstrates the comprehension of technical language used by the expert witness against how clear the juror thought the evidence was. A Man-Whitney test compared the juror’s opinion on their understanding of technical language with how clear they thought the image was. This took into account all three evidence types. The results demonstrate that visually, the clearer the jurors find the evidence and information depicted in the courtroom, the easier it is to understand the technical language used by the expert witness. This is validated by a significant *p* ≤ 0.001 and strongly suggests that clear images should be used in the courtroom, no matter what evidence visualisation technique is used.

**Table 5:** This demonstrates the understanding of technical language is enhanced by the clarity of the image

Understanding of technical language vs. image clarity	Mann-Whitney	*p* value
Yes	49.8	≤ 0.001
No	18.36	

## Discussion

Burns [3] suggested that images in the criminal legal process may bias in favour of the prosecution. In this study, this was not witnessed. However, there appears to be a change in perception when visualising 3D-printed models as demonstrative evidence because the guilty to not guilty ratio is more evenly split. It is possible that this is a factor of distraction. For instance, the majority of jurors who had a non-guilty verdict believed they had received insufficient evidence to state otherwise. On the other hand, the majority of jurors who stated guilty said their reasoning was due to the force required to break the cranium. The discussion on force was never mentioned in the mock trial, and therefore, the jurors established their own interpretation of the data. It is unknown as to whether this interpretation of the data is due to the sample group used within this study, and in future studies, a group that encompasses the wider population would be beneficial. However, this currently demonstrates that caution must be applied to the use of 3D-printed models and the authors believe that further research into the use of 3D-printed models should be undertaken prior to their continued use within the courtroom. This is reiterated in Blau et al.’s [18] study.

With regard to the clarity and understanding of the evidence, although not statistically significant, 98% of jurors stated they understood the terminology used when the 3D-printed models were utilised as demonstrative evidence, and 88% of jurors when visualising the 3D animations. This is an increase from the photographic method (79%) and this difference is echoed by Ampanozi et al. [17] who encourages the use of 3D reconstructions in forensic radiology reports. Similarly, this reiterates Blau et al. [18] who state that some visual methods are stronger for comprehension of evidence than others and therefore can be effective at communicating complex evidence. Likewise, Rutty et al. [21] also demonstrated an increase in understanding in nurse education with the use of innovative teaching tools. This is important because evidence that may be misinterpreted can have a drastic outcome on the final verdict, and using 3D imaging can reduce this concern because it increases the juror’s comprehension of evidence.

However, the outcome is not quite that straightforward. In cases where the evidence is deemed clear by the jurors, then comprehension of technical language shows statistical significance. This suggests that the overriding influence on the juror is the evidence type itself as opposed to the language associated with it. Therefore, since imaging modality does not influence juror decision-making, we recommend the use of 3D imaging since, if this is used for all evidence types, greater technical understanding can be achieved.

It may be argued that a limitation of this research is that the scenario is not realistic due to the use of an archaeological cranium to replicate an incident. This should be a consideration for future studies and perhaps clinical radiographs could be used. Alternatively, an archaeological cranium could represent data in a forensic anthropological context. Similarly, the results may demonstrate a sample bias because the majority of participants in this study were students. However, research on this topic has confirmed that this is not a cause for concern [22, 23].

## Conclusion

Several authors have stated that evidential findings must be presented to the court in a palatable and clear manner, while not compromising the integrity of the evidence displayed [17, 18]. The use of 3D imaging modalities in the courtroom is the current manifestation of illustrative evidence in a long timeline of evidential graphics. This research has demonstrated that the use of 3D imaging technology offers practical advantages to the courtroom, specifically with respect to the juror’s understanding of technical language. The results show that juror comprehension increases with the use of 3D digitisations and 3D-printed replicas. On the other hand, caution must be applied to the use of 3D-printed models before additional research has been undertaken. Therefore, the authors encourage further research into comparisons between how images and 3D-printed models can evoke emotional responses, the use of 3D imaging in court, the decision-making process, and 3D-printed models for further understanding of this topic.

## References

[1] Morgan RM, Bull PA (2007). Forensic geoscience and crime detection. Identification, interpretation and presentation in forensic geoscience. Minerva Med.

[2] Burgoon JK, Bonito JA, Bengtsson B, Cederberg C, Lundeberg M, Allspach L (2000). Interactivity in human-computer: a study of credibility, understanding, and influence. Comput Hum Behav.

[3] Burns D (2001). When used in the criminal legal process forensic science shows a bias in favour of the prosecution. Sci Justice.

[4] March J, Schofield D, Evison M, Woodford N (2004) Three-dimensional computer visualization of forensic pathology data. Am J Forensic Med 60–70 10.1097/01.paf.0000113863.69360.42 15075692

[5] Durnal EW (2010). Crime scene investigations (as seen on TV). Forensic Sci Int.

[6] Nakhaeizadeh S, Dror I, Morgan R (2014). Cognitive bias in forensic anthropology: visual assessment of skeletal remains is susceptible to confirmation bias. Sci Justice.

[7] Errickson D, Thompson TJU, Rankin BWJ (2014). The application of 3D visualization of osteological trauma for the courtroom: a critical review. Journal of Forensic Radiology and Imaging.

[8] Thompson TJU (2008). The role of the photograph in the application of forensic anthropology and the interpretation of clandestine scenes of crime. Photogr Cult.

[9] Carew RM, Errickson D (2019). Imaging in forensic science: five years on. Journal of Forensic Radiology and Imaging.

[10] Thompson T, Norris P (2018). A new method for the recovery and evidential comparison of footwear impressions using 3D structured light scanning. Sci Justice.

[11] Shamata A, Thompson T (2018). Documentation and analysis of traumatic injuries in clinical forensic medicine involving structured light three-dimensional surface scanning versus photography. J Forensic Legal Med.

[12] Buck U, Naether S, Braun M, Bolliger S, Friederich H, Jackowski C, Aghaye E, Christie A, Vock P, Dirnhofer R, Thali MJ (2007). Application of 3D documentation and geometric reconstruction methods in traffic accident analysis: with high resolution surface scanning, radiological MSCT/MRI scanning and real data based animation. Forensic Sci Int.

[13] Clifford M, Kinloch K (2007). The use of computer simulation evidence in court. Comp Law Secur Rev.

[14] Grassberger M, Gehl A, Püschel K, Turk EE (2011). 3D reconstruction of emergency cranial computed tomography scans as a tool in clinical forensic radiology after survived blunt head trauma – report of two cases. Forensic Sci Int.

[15] de Bakker BS, Soerdjbalie-Maikoe V, De Bakker HM (2013). The use of 3D-CT in weapon caused impression fractures of the skull, from a forensic radiological point of view. J Forensic Radiol Imaging.

[16] Flemming-Farrell D, Michaildis K, Karantanas A, Roberts N, Kranioti EF (2013). Virtual assessment of perimortem and post-mortem blunt force cranial trauma. Forensic Sci Int.

[17] Ampanozi G, Zimmermann D, Hatch GM, Ruder TD, Ross S, Flach PM, Thali MJ, Ebert LC (2012). Format preferences of district attorneys for post-mortem medical imaging reports: understandability, cost effectiveness, and suitability for the courtroom: a questionnaire based study. Legal Med.

[18] Blau S, Phillips E, O’Donnell C, Markowsky G (2018) Evaluating the impact of different formats in the presentation of trauma evidence in court: a pilot study. Austr J Forensic Sci. 10.1080/00450618.2018.1457717

[19] Taylor E, Wolframm-Murray Y, Yates A (2011) Archaeological excavation at Stanground South Peterborough: assessment report and updated project design, Northamptonshire Archaeology, Unpublished Report 11/01

[20] Errickson D, Thompson TJ, Rankin BW (2015) An optimum guide for the reduction of noise using a surface scanner for digitising human osteological remains. [online] Available at: http://guides.archaeologydataservice.ac.uk/g2gp/CS_StructuredLight. Accessed 4 Oct 2018

[21] Rutty J, Biggs M, Dowsett D, Kitchener A, Coltman N, Rutty G (2019). Post mortem computed tomography: an innovative tool for teaching anatomy within pre-registration nursing curricula. Nurse Educ Today.

[22] Bornstein BH, Neuschatz J, Magyarics C, Golding JM, Kimbrough C, Reed K, Luecht K (2017). Mock juror sampling issues in jury simulations research: a meta-analysis. Law Hum Behav.

[23] Lieberman JD, Krauss DA, Heen M, Sakiyama M (2016). The good, the bad, and the ugly: professional perceptions of jury decision-making research practices. Behav Sci Law.

